# Supplementary material for: High-Speed Videoendoscopy Enhances the Objective Assessment of Glottic Organic Lesions: A Case-Control Study with Multivariable Data-Mining Model Development
Source: Cancers (Basel). 2023 Jul 22;15(14):3716. doi: 10.3390/cancers15143716 (PMC10378075; doi:10.3390/cancers15143716)
Supplement: Supplementary file 1 [file cancers-15-03716-s001.zip › cancers-2434700-supplementary File S1.pdf]

Normophonic vs any organic lesion

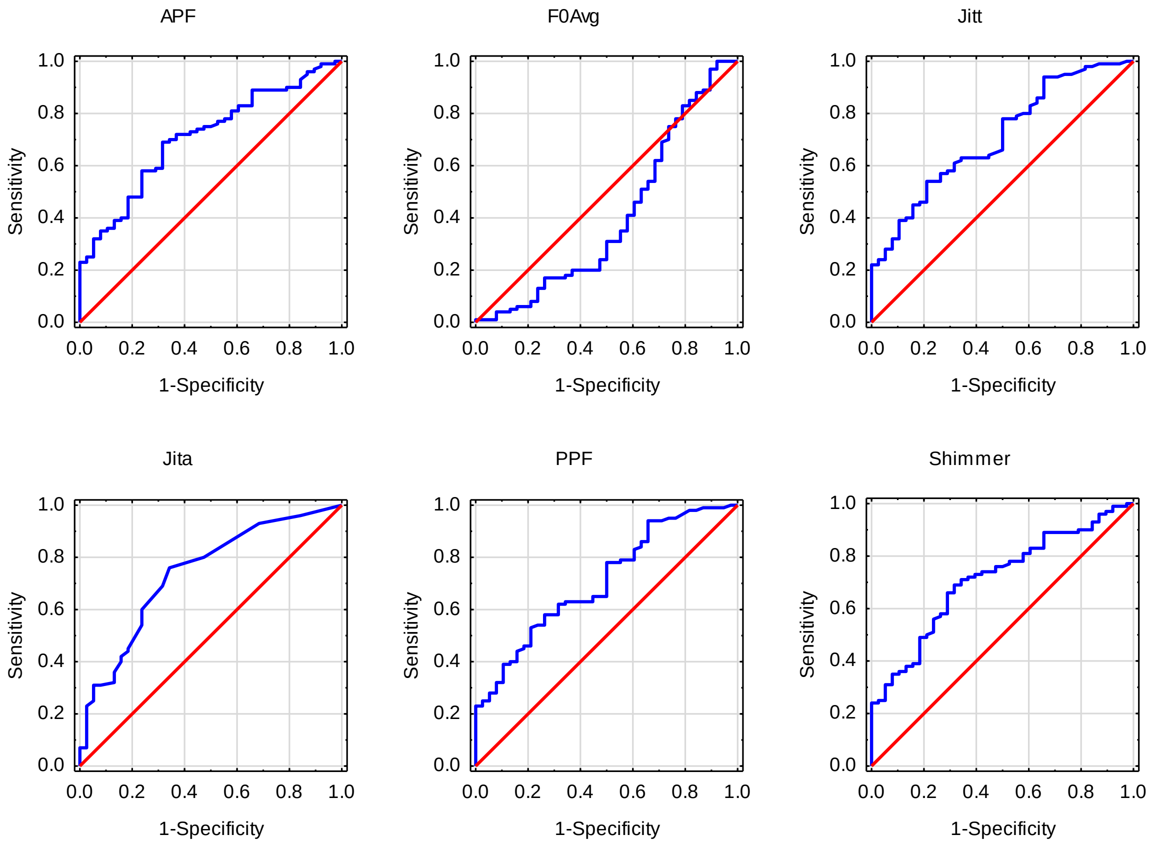

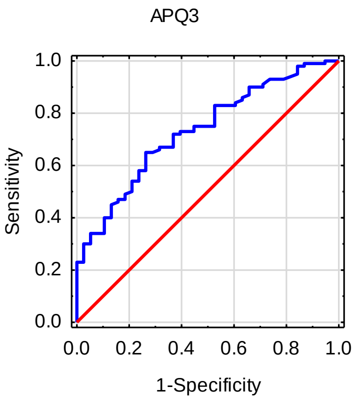

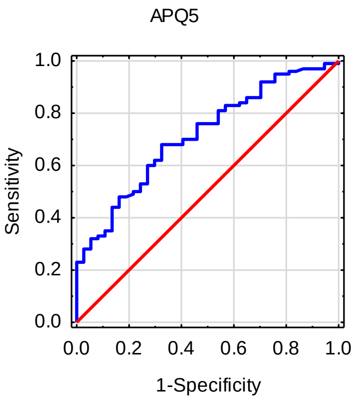

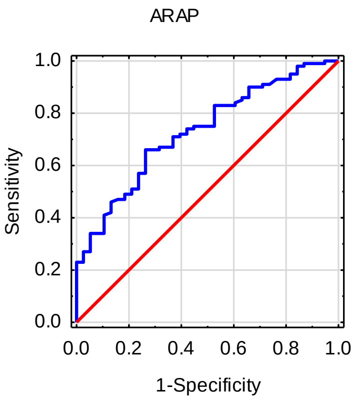

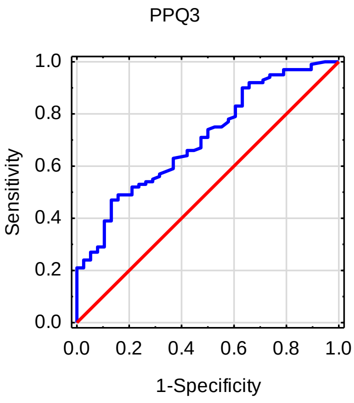

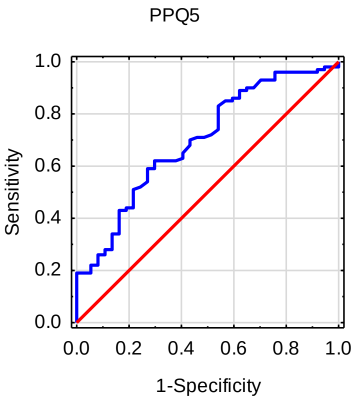

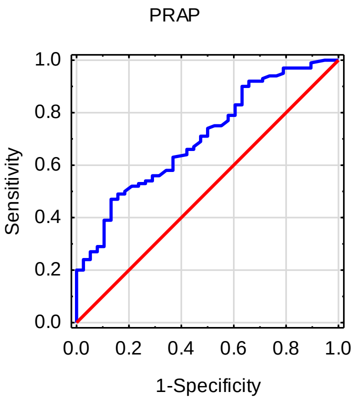

	Zmienna: Jita [ms]_2					
	AUC	SE	AUC Dolny 95%	AUC Górny 95%	z	p
1	0.739	0.047	0.646	0.832	5.036	0.0000

	Zmienna: APQ3 [%]_2					
	AUC	SE	AUC Dolny 95%	AUC Górny 95%	z	p
1	0.733	0.045	0.644	0.821	5.161	0.0000

	Zmienna: ARAP [%]_2					
	AUC	SE	AUC Dolny 95%	AUC Górny 95%	z	p
1	0.731	0.045	0.642	0.819	5.099	0.0000

	Zmienna: APQ5 [%]_2					
	AUC	SE	AUC Dolny 95%	AUC Górny 95%	z	p
1	0.715	0.047	0.624	0.807	4.616	0.0000

	Zmienna: Shimmer [%]_2					
	AUC	SE	AUC Dolny 95%	AUC Górny 95%	z	p
1	0.712	0.046	0.621	0.803	4.577	0.0000

	Zmienna: APF [%]_2					
	AUC	SE	AUC Dolny 95%	AUC Górny 95%	z	p
1	0.709	0.046	0.618	0.8	4.499	0.0000

	Zmienna: PPF [%]_2					
	AUC	SE	AUC Dolny 95%	AUC Górny 95%	z	p
1	0.708	0.047	0.615	0.801	4.392	0.0000

	Zmienna: Jitt [%]_2					
	AUC	SE	AUC Dolny 95%	AUC Górny 95%	z	p
1	0.708	0.048	0.615	0.801	4.372	0.0000

	Zmienna: PRAP [%]_2					
	AUC	SE	AUC Dolny 95%	AUC Górny 95%	z	p
1	0.705	0.048	0.612	0.798	4.312	0.0000

	Zmienna: PPQ3 [%]_2					
	AUC	SE	AUC Dolny 95%	AUC Górny 95%	z	p
1	0.705	0.048	0.611	0.798	4.305	0.0000

	Zmienna: PPQ5 [%]_2					
	AUC	SE	AUC Dolny 95%	AUC Górny 95%	z	p
1	0.697	0.05	0.6	0.795	3.963	0.0001

	Zmienna: F0Avg [Hz]_2					
	AUC	SE	AUC Dolny 95%	AUC Górny 95%	z	p
1	0.405	0.06	0.288	0.521	-1.6	0.1096

Benign vs Malignant organic lesion

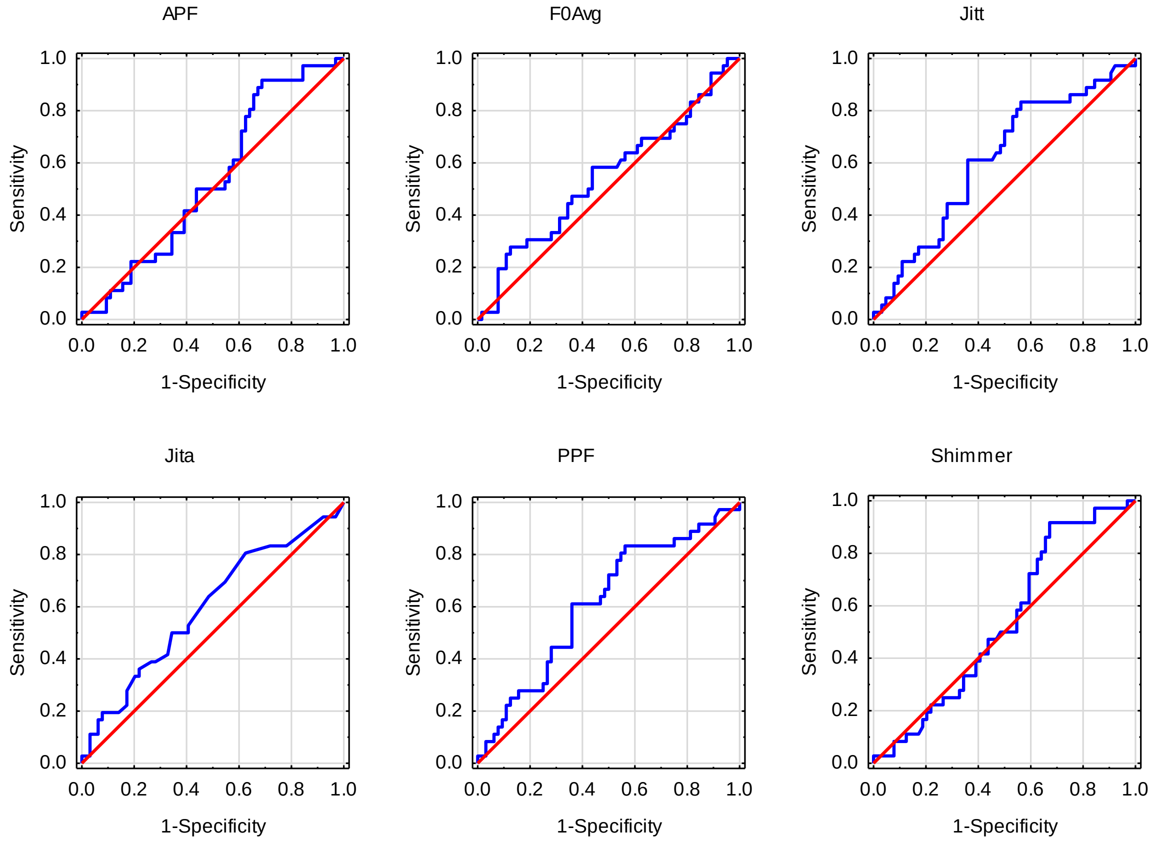

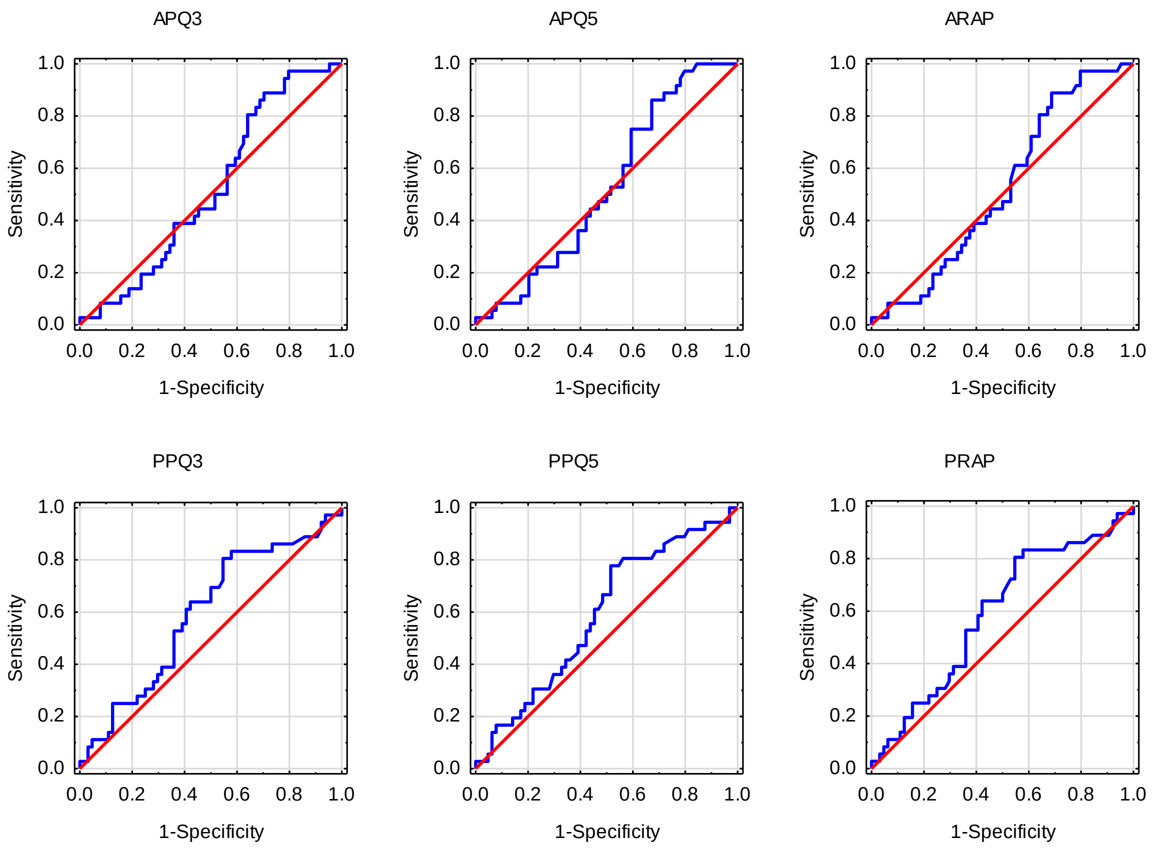

	Zmienna: PPF [%]_2					
	AUC	SE	AUC Dolny 95%	AUC Górny 95%	z	p
1	0.613	0.058	0.499	0.728	1.942	0.0521

	Zmienna: Jitt [%]_2					
	AUC	SE	AUC Dolny 95%	AUC Górny 95%	z	p
1	0.612	0.058	0.497	0.726	1.91	0.0561

	Zmienna: Jita [ms]_2					
	AUC	SE	AUC Dolny 95%	AUC Górny 95%	z	p
1	0.596	0.06	0.479	0.712	1.606	0.1082

	Zmienna: PPQ3 [%]_2					
	AUC	SE	AUC Dolny 95%	AUC Górny 95%	z	p
1	0.594	0.059	0.479	0.71	1.599	0.1099

	Zmienna: PRAP [%]_2					
	AUC	SE	AUC Dolny 95%	AUC Górny 95%	z	p
1	0.591	0.059	0.475	0.706	1.542	0.1230

	Zmienna: PPQ5 [%]_2					
	AUC	SE	AUC Dolny 95%	AUC Górny 95%	z	p
1	0.589	0.058	0.475	0.704	1.529	0.1262

	Zmienna: F0Avg [Hz]_2					
	AUC	SE	AUC Dolny 95%	AUC Górny 95%	z	p
1	0.546	0.062	0.425	0.666	0.744	0.4567

	Zmienna: Shimmer [%]_2					
	AUC	SE	AUC Dolny 95%	AUC Górny 95%	z	p
1	0.537	0.058	0.423	0.65	0.637	0.5244

	Zmienna: APF [%]_2					
	AUC	SE	AUC Dolny 95%	AUC Górny 95%	z	p
1	0.536	0.058	0.422	0.649	0.613	0.5401

	Zmienna: APQ5 [%]_2					
	AUC	SE	AUC Dolny 95%	AUC Górny 95%	z	p
1	0.529	0.058	0.416	0.642	0.505	0.6136

	Zmienna: ARAP [%]_2					
	AUC	SE	AUC Dolny 95%	AUC Górny 95%	z	p
1	0.525	0.058	0.412	0.638	0.432	0.6658

	Zmienna: APQ3 [%]_2					
	AUC	SE	AUC Dolny 95%	AUC Górny 95%	z	p
1	0.521	0.058	0.408	0.635	0.371	0.7105
